# Effect of an armed conflict on relative socioeconomic position of rural households: case study from western Côte d'Ivoire

**DOI:** 10.1186/1742-7622-7-6

**Published:** 2010-08-31

**Authors:** Thomas Fürst, Andres B Tschannen, Giovanna Raso, Cinthia A Acka, Don de Savigny, Olivier Girardin, Eliézer K N'Goran, Jürg Utzinger

**Affiliations:** 1 Department of Epidemiology and Public Health, Swiss Tropical and Public Health Institute, Basel, Switzerland; 2 University of Basel, Basel, Switzerland; 3 Centre Suisse de Recherches Scientifiques en Côte d'Ivoire, Abidjan, Côte d'Ivoire; 4 Molecular Parasitology Laboratory, Queensland Institute of Medical Research, Brisbane, Australia; 5 School of Population Health, University of Queensland, Brisbane, Australia; 6 Département de Sociologie, Université de Cocody-Abidjan, Abidjan, Côte d'Ivoire; 7 Fondation Rurale Interjurassienne, Courtemelon, Courtételle, Switzerland; 8 UFR Biosciences, Université de Cocody-Abidjan, Abidjan, Côte d'Ivoire

## Abstract

**Background:**

Current conceptual frameworks on the interrelationship between armed conflict and poverty are based primarily on aggregated macro-level data and/or qualitative evidence and usually focus on adherents of warring factions. In contrast, there is a paucity of quantitative studies about the socioeconomic consequences of armed conflict at the micro-level, i.e., noncommitted local households and civilians.

**Methods:**

We conducted a secondary analysis of data pertaining to risk factors for malaria and neglected tropical diseases. Standardized questionnaires were administered to 182 households in a rural part of western Côte d'Ivoire in August 2002 and again in early 2004. Between the two surveys, the area was subject to intensive fighting in the Ivorian civil war. Principal component analysis was applied at the two time points for constructing an asset-based wealth-index and categorizing the households in wealth quintiles. Based on quintile changes, the households were labeled as 'worse-off', 'even' or 'better-off'. Statistical analysis tested for significant associations between the socioeconomic fates of households and head of household characteristics, household composition, village characteristics and self-reported events associated with the armed conflict. Most-poor/least-poor ratios and concentration indices were calculated to assess equity changes in households' asset possession.

**Results:**

Of 203 households initially included in the first survey, 21 were lost to follow-up. The population in the remaining 182 households shrunk from 1,749 to 1,625 persons due to migration and natural population changes. However, only weak socioeconomic dynamics were observed; every seventh household was defined as 'worse-off' or 'better-off' despite the war-time circumstances. Analysis of other reported demographic and economic characteristics did not clearly identify more or less resilient households, and only subtle equity shifts were noted.

However, the results indicate significant changes in livelihood strategies with a significant return to agricultural production and a decrease in the diversity of socioeconomic activities.

**Conclusion:**

Situational constraints and methodological obstacles are inherent in conflict settings and hamper conflict-related socioeconomic research. Furthermore, sensitive methods to assess and meaningfully interpret longitudinal micro-level wealth data from low-income countries are lacking. Despite compelling evidence of socioeconomic dynamics triggered by armed conflicts at the macro-level, we could not identify similar effects at the micro-level. A deeper understanding of household profiles that are more resilient to armed conflict could help to better prevent and/or alleviate adverse conflict-related and increasingly civilian-borne socioeconomic effects.

## Introduction

The importance of local, national and international efforts to halve extreme poverty on a global scale by 2015–one of the eight United Nations (UN) Millennium Development Goals (MDGs)–is partially driven by poverty's spurring effect on armed conflict and war (see endnote 1).Simultaneously, armed conflict and war have an undisputed effect on poverty itself. Consequently, poverty is a *cause *and a *consequence *of armed conflict and war.

Current conceptual frameworks for analyzing the interrelationship between armed conflict and war and poverty are mainly based on three legs; namely (i) cost of conflict, (ii) grievance and (iii) greed. The idea that armed conflict and war induce broad socioeconomic losses, and hence exacerbate poverty is widely accepted and supported by various cost of conflict analyses [1]. Cost of conflict analyses range from direct economic losses due to decreased growth rates of a nation's gross domestic product (GDP) [2-5] to direct and indirect costs on human health [6-12].

Additionally, there are less apparent costs due to human rights violations [13] and ecological damage [14,15].

There is less agreement, however, about the reverse theory: poverty *causing *armed conflict and war [1]. According to the grievance approach, unbalanced societal development leads to inequality, exclusion, and poverty, which in turn contribute to growing grievances that might lead to violent conflict [1,16-18]. Recent economic research challenges this grievance approach, as it might oversimplify the reality [19]. Neoclassical economic theory stresses that there are not only costs incurred by armed conflict and war, but also benefits, at least for certain population groups. Supporters of this theory argue that rather than just grievance, opportunities for predatory accumulation–namely greed–tends to cause conflict and war [20-23].

The aforementioned concepts are based principally on aggregated macro-level and/or qualitative evidence and focus on adherents of warring factions. Despite a rich body of literature on collateral micro-level health consequences of armed conflict [24-26], quantitative studies about the socioeconomic consequences of armed conflict and war at the micro-level (e.g. on trapped local households and non-committed civilians) are scarce [27]. In a recent article, we highlighted how armed conflict, health and wealth may be interlinked with the physical, social and socioeconomic environment [28]. An additional issue is that–while methodological difficulties regarding the interpretation of longitudinal socioeconomic micro-level data remain–theories aimed at the macro-level lack a solid foundation without sound micro-level data, and important micro-level information remains neglected. For example, both causes and consequences of armed conflict and war are not shared equally amongst adherents of warring factions or non-committed civilians over an entire country and a longer period.

In order to contribute to a better understanding of micro-level socioeconomic consequences of armed conflict and war on civilians, we took the rare opportunity to have access to reliable longitudinal data on households located in a region of armed conflict in rural western Côte d'Ivoire. In a secondary analysis of two datasets, one obtained prior to the armed conflict and the other after military hostilities had ended, we applied the standard technique of principal component analysis (PCA) to construct an asset-based wealth index and investigate the impact of the armed conflict on relative socioeconomic position (SEP) of the households. Based on the results, we identified 'better-off', 'even' and 'worse-off' households. This outcome was then compared with other household characteristics and statements about the most important problems encountered during the conflict in order to check for distinct qualities of households that were socioeconomically more or less successful. Furthermore, concentration indices (CIs) and most poor/least poor (MP/LP) ratios were calculated to analyze changes in asset accumulation. We expected strong dynamics in socioeconomic indicators and this hypothesis is discussed in the light of the study design, methods and key findings.

## Methods

### Armed conflict in Côte d'Ivoire

In recent years, West Africa has witnessed growing political unrest, armed conflict, civil war and riots. Côte d'Ivoire, the economic powerhouse of the West African Monetary Union (UMEOA), has suffered increasingly violent conflict, starting with a coup d'état towards the end of 1999 that proceeded to a full-fledged armed conflict in 2002. This armed conflict officially ended in July 2003, but violence still flared up occasionally (e.g. during a break of cease-fire in November 2004). This tumultuous period left the country divided into a rebel-held North and a government-held South. The conflict had far reaching political implications and its resolution was on the agenda of international stakeholders, including the UN, the African Union (AU), France and a host of African presidents [29].

While the origins of the armed conflict in Côte d'Ivoire are still debated, possible causes include problems related to government services, legal issues about land tenure and other scarce natural resources, political bias on eligibility to stand for election, ethnic discrimination and racism [30-33]. Several attempts have been made to study the consequences of the Ivorian conflict. For example, it is estimated that foreign direct investments in Côte d'Ivoire dropped by more than 60% (roughly 100 billion Franc CFA) during the sociopolitical crisis, which preceded the armed conflict [34]. The subsequent armed conflict left many research efforts in tatters [35], led to approximately 750,000 internally displaced persons (IDPs) and 500,000 refugees [36], caused a reduction in health staff of over 75% and an abandonment of health facilities of 80% [37].

### Study area

The present study was carried out in the region of Man, western Côte d'Ivoire, between August 2002 and February 2004. The area belongs to the Ivorian rainforest zone, which is part of the Western African forest belt, but the forests have come under increased pressure of exploitation over the last decades [38,39].

Before the outbreak of the 2002 armed conflict, about 250,000 people lived in the region of Man, with approximately half living in the regional capital Man [40-42]. Local rural dwellers were mainly engaged in subsistence farming, cultivating upland and paddy rice, cassava, maize, plantain and yam. The most important cash crops were rice, coffee and cocoa, and the region also had a small timber industry [40]. Additionally, goats, cattle and poultry were kept as meat animals and these food sources were complemented by inland fish farming [42,43]. During the armed conflict, the area belonged to the rebel-held North and was subjected to intensive fighting as it was close to the military frontlines. The conflict had a deteriorating effect on the regional economy and there were reports of looting (e.g. the local fingerling production). In one survey, the percentage of households mentioning a permanent workplace decreased from 85% to 69% [44]. The population's vulnerability in terms of nutrition increased; household's reported disposable daily food budgets were almost halved and the number of meals per day dropped from an average of 2.5 to 1.7 [44]. Also, substantial forced migration occurred in the area, as estimates suggest every eighth person was displaced [44], and some villages were temporarily cut off from the outside world [45]. Schools were closed and physical infrastructure destroyed [37,45]. Public health programs in western Côte d'Ivoire suffered disproportionately with a loss of 88% of all trained health staff and 90% of all health facilities [37].

### Questionnaire-based data collection

Since the mid-1990s, the Centre Suisse de Recherches Scientifiques en Côte d'Ivoire (CSRS) and the Université de Cocody-Abidjan–in collaboration with the Swiss Tropical and Public Health Institute (Swiss TPH)–has carried out research and integrated control of infectious diseases (mainly malaria, schistosomiasis and soil-transmitted helminthiasis) in the study area (see endnote 2). These efforts have led to the development of a comprehensive database that, created before the armed conflict erupted, offered a unique baseline for any subsequent war-related impact analyses at the micro-level.

Baseline data for the present study were obtained from a household-based survey that was initially designed for assessing risk factors for malaria, schistosomiasis and soil-transmitted helminthiasis. This first household survey was conducted in August 2002, just one month before the armed conflict erupted. For data collection, a standardized questionnaire was developed, pretested and then administered by trained assistants who interviewed the respective heads of randomly selected households. The questionnaire included sections about general household characteristics (e.g. head of households' demographics and educational level; number, age and sex of all household members; main source of household income), health, hygiene, environmental risk factors, use of information media and a socioeconomic part comprising an asset list for the calculation of wealth indices. The asset list consisted of information on different household assets, including dwelling characteristics, house and land possession, electronic devices, sanitary and personal protective products (e.g. insecticide-treated bednets) and transport means. Notably lacking were questions related to investment assets and public goods other than access to the power grid. Prior to this study, similar asset lists have been used for evaluating the socioeconomic status in the study area [41,42,46-49].

After an official ceasefire in July 2003, researchers returned to the Man region under the auspices of a new project to integrate existing knowledge on intestinal parasites, but also to attempt to further the peace process and national reconciliation at a grass-roots level. The project was complemented by humanitarian aid activities through the provision of village and/or school-level first-aid kits and deworming of approximately 130,000 school-aged children and other high-risk groups (see endnote 3).

During this process, the same households from the previously mentioned August 2002 survey were traced according to (i) location and (ii) household members. In order to obtain informed consent for the secondary analysis, the purpose and procedures of the follow-up survey were discussed with local authorities and study participants. As the majority of the study participants were illiterate, oral informed consent was sought in the presence of district and regional health and education authorities, with detailed explanations given in the local language by trained field assistants and local witnesses. This is the usual procedure when administering questionnaires without concurrent collection of biological samples (e.g. blood, stool or urine) in Côte d'Ivoire, and the whole process was approved by the institutional research commissions of CSRS and Swiss TPH and cleared by the national health and education authorities of Côte d'Ivoire. Subsequently, the consenting heads of households were interviewed again and, in order to allow for longitudinal comparison in a secondary analysis, were asked exactly the same questions as in the first survey. Additional questions about births, deaths, migration and the impact of the conflict were included in the second survey.

### Operational details and community description

Operational details and a description of participating households' demographic profiles have been presented elsewhere (see endnote 4). In brief, the first survey included 7-9 randomly selected households from each of the 25 villages in the region of Man (total 203 households). This sample size represented approximately 5-10% of all households in the respective villages. The 25 villages were located within 4-40 km air-line distance around the regional capital of Man. Ten villages were accessible via main streets, 7 via minor streets and 8 only via gravel roads. Population estimates for the 25 villages ranged from 200 to 2,000 individuals. Interviews indicated that subsistence farming and agricultural production for markets were the two most important economic activities in all villages before the armed conflict, which is in accordance with the previously described regional economic structure. In addition, trade and fishing were other important activities in 10 villages, wood processing in 4 villages and artisanry in one village.

**Figure 1:**
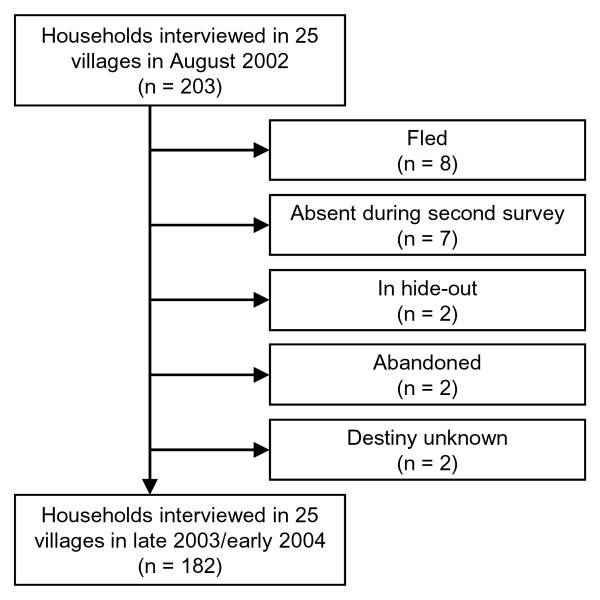
**Operational results and the final study sample of the two surveys carried out in the Man region of western Côte d'Ivoire in August 2002 and late 2003/early 2004**.

Between the first and the second survey and based on information from their neighbors, the members of 8 households fled because of the armed conflict, 7 households remained but their members were absent during the second survey, and 2 households each were in hide-out near the village, abandoned, or disappeared with unknown destiny. Consequently, the final study sample consisted of 182 households in 25 villages that have been interviewed twice (see Figure 1).

The number of inhabitants of the 182 studied households decreased from 1,749 to 1,625 between the first and second survey. This decrease was due to a negative migration balance (n = -74) and a negative natural population change (n = -59). These figures include an error due to misreporting of +9 individuals, which equals 0.5% of the original population. According to the heads of households, 66% of the absent household members had migrated to urban settings nearby or farther away. Furthermore, our data implied a lowered birth rate and an increased death rate. However, age and sex distribution did not vary significantly between the two surveys, and we can only speculate about the impact of the armed conflict on these findings (see Table 1 and endnote 4).

**Table 1:** Population, migration and natural population changes in the 182 households included in the August 2002 and late 2003/early 2004 surveys, stratified by sex and two age groups.

Demographic characteristics	Total no.	% of original population	Sex ratio male:female	% under 16 years
Population 1^st ^survey (August 2002)	1,749	100.0	1.05	50.3
				
Migration				
Immigration	+64	+3.7	1.13	26.6
Emigration	-138	-7.9	1.23	39.1
				
Natural population changes				
Births	+36	+2.0	1.25	---
Deaths	-95	-5.4	1.07	25.3
				
Population 2^nd ^survey (late 2003/early 2004)	1,625^a^	92.9^a^	1.07	52.6
				
Population change	-124	-7.1	+0.02	+2.3

^a ^Includes a misreporting of +9 persons, which equals +0.5% of the original population

### Analyses

Questionnaire data were double entered and cross-checked using EpiInfo v.6.04 (Centers for Disease Control and Prevention; Atlanta, GA, USA). Statistical analyses were performed with STATA v.9.1 (STATA Corporation; College Station, TX, USA). All statistical tests were carried out at a 5% significance level.

Data analysis was restricted to households with both complete baseline and follow-up data. For calculating a household's socioeconomic status, an asset-based index was constructed for each survey separately, according to a method described by Filmer and Pritchett [50]. In short, the applied method assigns a weighted sum as a proxy for socioeconomic wealth to each household, based on the following formula (1):

(1) $${\text{A}}_{j}\;\text{=}\;\sum_{k}\left[\left(a_{jk}-{\bar{a}}_{k}\right)/s_{k}*f_{k}\right]$$

*A_j _*is the asset score of household *j*, *a_jk _*is a binary variable of asset *k *for household *j *(0 = household does not have asset; 1 = household has asset; the only exception is the number of people per sleeping room where decimal numbers were used), *ā_k _*and *s_k _*are the unweighted sample mean and standard deviation (SD) of asset *k *over all households and, finally, *f_k _*is the so called 'raw' asset factor of asset *k*. This 'raw' asset factor is defined as the first component of the eigenvector of the respective asset and is derived by using PCA. According to formula (1), the possession of an asset increases *A_j _*if the asset's eigenvector is positive. Hence, a positive eigenvector implies that an asset is a 'good' and its possession is associated with a higher SEP. In case of a negative eigenvector, an asset is considered a 'bad' and its possession is associated with a lower SEP. This method allows for ranking of the households according to their achieved weighted sum, *A_j_*, in the two surveys and subsequent division into wealth quintiles. The whole procedure is further explained and illustrated in technical notes provided by the HNP/Poverty Thematic Group of the World Bank [51] and elsewhere [52] (see endnote 5).

For longitudinal analysis of socioeconomic wealth changes, households which are scaled down by more than one quintile between the two surveys were labeled as 'worse-off' and households which are scaled up by more than one quintile as 'better-off'. Fisher's exact test, Kruskal-Wallis test, and McNemar's χ² test were used as appropriate to find significant associations between the households' socioeconomic fates and other characteristics that were obtained by questionnaires.

MP/LP ratios and CIs were calculated in order to measure equity changes in households' asset possession [53,54]. Computation and analysis of CIs were carried out according to the convenient covariance and the convenient regression method for the micro-data case as described elsewhere [52] (see endnote 5).

All first survey characteristics of the 21 households lost to follow-up were compared with those of the 182 re-identified households in an attrition analysis. No significant differences were found, and hence potential selection bias considered as unlikely.

## Results

### Assets and asset score characteristics

Tables 2 and 3 give an overview of the asset possession of the 182 households in each survey, the calculated unweighted means and SD, the first component of the eigenvectors, and the scores obtained. Moreover, they show the distribution of all included assets over the computed wealth quintiles.

**Table 2:** Overview of included assets, household asset possession and asset scores in the first survey (before the armed conflict in Côte d'Ivoire, August 2002).

Asset variable	Proportion of households possessing the asset						Mean (ā_k_)	SD (s_k_)	Eigenvector (f_k_)	Household score if household	
	Wealth quintiles										
						
	Most poor	Very poor	Poor	Less poor	Least poor	Total	Unweighted		First component (='raw' asset factor)	Has asset	Does not have asset
Possession of land	0.97	0.97	0.95	0.92	0.81	0.92	0.923	0.267	-0.1024	-0.0295	0.3538
Possession of house	1.00	0.94	0.97	0.97	0.92	0.96	0.962	0.193	-0.0463	-0.0092	0.2309
People per sleeping room^a^	4.5^a^	4.3^a^	2.5^a^	2.6^a^	2.9^a^	2.7^a^	2.662	2.255	-0.1580	---^b^	---^b^
Type of wall											
Wood	0.05	0.00	0.00	0.00	0.00	0.01	0.011	0.105	-0.1021	-0.9663	0.0107
Mud/clay	0.87	0.33	0.27	0.25	0.00	0.35	0.346	0.477	-0.2723	-0.3731	0.1976
Cement	0.08	0.67	0.73	0.75	1.00	0.64	0.643	0.481	0.2926	0.2175	-0.3915
Type of roof											
Thatch	0.60	0.06	0.03	0.03	0.00	0.14	0.143	0.351	-0.2789	-0.6812	0.1136
Tiles	0.03	0.00	0.05	0.08	0.03	0.04	0.039	0.193	0.0424	0.2115	-0.0085
Corrugated sheets	0.38	0.94	0.92	0.89	0.97	0.82	0.819	0.386	0.2321	0.1089	-0.4919
Energy source for cooking											
Wood	1.00	0.97	1.00	0.97	0.94	0.98	0.978	0.147	-0.0606	-0.0091	0.4032
Coal	0.00	0.03	0.00	0.03	0.08	0.03	0.027	0.164	0.0782	0.4640	-0.0129
Electronic devices											
Electricity in house	0.05	0.17	0.49	0.86	1.00	0.51	0.511	0.501	0.3419	0.3335	-0.3485
Refrigerator	0.00	0.00	0.00	0.03	0.22	0.05	0.050	0.217	0.1376	0.6016	-0.0313
Radio	0.35	0.50	0.65	0.86	0.92	0.65	0.654	0.477	0.2211	0.1604	-0.3030
Television	0.00	0.00	0.14	0.36	0.86	0.27	0.269	0.445	0.3287	0.5400	-0.1989
Ventilator	0.00	0.00	0.03	0.11	0.44	0.12	0.115	0.320	0.2143	0.5917	-0.0772
Video	0.00	0.00	0.00	0.00	0.03	0.01	0.005	0.074	0.0633	0.8511	-0.0043
Sanitation infrastructure and personal protection											
No toilet	0.73	0.81	0.19	0.14	0.11	0.40	0.396	0.490	-0.2762	-0.3405	0.2232
Uncemented toilet	0.16	0.11	0.11	0.03	0.06	0.09	0.093	0.292	-0.0693	-0.2153	0.0221
Cemented toilet	0.11	0.08	0.68	0.81	0.81	0.49	0.494	0.501	0.3028	0.3058	-0.2986
Soap	1.00	0.97	1.00	1.00	0.97	0.99	0.989	0.105	0.0189	0.0020	-0.1780
Insecticide spray	0.11	0.06	0.05	0.39	0.39	0.20	0.198	0.399	0.1516	0.3047	-0.0752
Mosquito net	0.16	0.22	0.19	0.19	0.39	0.23	0.231	0.422	0.0743	0.1354	-0.0407
Main mean of transportation											
Feet/walking	1.00	0.97	0.84	0.67	0.50	0.80	0.797	0.404	-0.2331	-0.1174	0.4601
Bicycle	0.00	0.03	0.03	0.25	0.33	0.13	0.126	0.333	0.1985	0.5204	-0.0753
Car/motorbike	0.00	0.00	0.14	0.08	0.17	0.08	0.077	0.267	0.1044	0.3607	-0.0300
											
Number of households	37	36	37	36	36	182					

^a ^Reports the average number of people per sleeping room in the respective wealth quintile

^b ^Computed score for one additional person per sleeping room: -0.0701

**Table 3:** Overview of included assets, household asset possession and asset scores in the second survey (after the armed conflict in Côte d'Ivoire, late 2003/early 2004).

Asset variable	Proportion of households possessing the asset						Mean (ā_k_)	SD (s_k_)	Eigenvector (f_k_)	Household score if household	
	Wealth quintiles										
						
	Most poor	Very poor	Poor	Less poor	Least poor	Total	Unweighted		First component (='raw' asset factor)	Has asset	Does not have asset
Possession of land	0.97	1.00	1.00	1.00	0.86	0.97	0.967	0.179	-0.1590	-0.0293	0.8590
Possession of house	0.97	1.00	1.00	0.97	0.86	0.96	0.962	0.193	-0.1403	-0.0280	0.6997
People per sleeping room^a^	3.9^a^	4.4^a^	2.4^a^	2.6^a^	3.1^a^	2.7^a^	2.663	2.508	-0.0582	---^b^	---^b^
Type of wall											
Wood	0.05	0.03	0.00	0.00	0.00	0.02	0.017	0.128	-0.0659	-0.5075	0.0085
Mud/clay	0.78	0.28	0.14	0.03	0.00	0.25	0.247	0.433	-0.2679	-0.4661	0.1531
Cement	0.16	0.69	0.87	0.97	1.00	0.74	0.736	0.442	0.2813	0.1679	-0.4687
Type of roof											
Thatch	0.51	0.17	0.00	0.00	0.00	0.14	0.137	0.345	-0.2176	-0.5437	0.0866
Tiles	0.03	0.08	0.08	0.03	0.03	0.05	0.050	0.217	-0.0094	-0.0411	0.0021
Corrugated sheets	0.46	0.75	0.92	0.97	0.97	0.81	0.813	0.391	0.1975	0.0944	-0.4110
Energy source for cooking											
Wood	1.00	0.97	0.97	1.00	0.92	0.97	0.973	0.164	-0.1405	-0.0231	0.8336
Coal	0.00	0.03	0.03	0.00	0.06	0.02	0.022	0.147	0.0990	0.6587	-0.0148
Electronic devices											
Electricity in house	0.16	0.31	0.54	0.83	0.89	0.54	0.544	0.499	0.2510	0.2292	-0.2734
Refrigerator	0.00	0.00	0.00	0.00	0.33	0.07	0.066	0.249	0.2047	0.7682	-0.0542
Radio	0.22	0.39	0.43	0.78	1.00	0.56	0.560	0.498	0.2582	0.2281	-0.2907
Television	0.00	0.00	0.14	0.31	0.81	0.25	0.247	0.433	0.3151	0.5483	-0.1801
Ventilator	0.00	0.00	0.03	0.11	0.50	0.13	0.126	0.333	0.2680	0.7027	-0.1017
Video	0.00	0.00	0.00	0.00	0.14	0.03	0.027	0.164	0.2122	1.2590	-0.0349
Sanitation infrastructure and personal protection											
No toilet	0.95	0.75	0.62	0.22	0.08	0.53	0.527	0.501	-0.2719	-0.2567	0.2860
Uncemented toilet	0.05	0.08	0.16	0.22	0.14	0.13	0.132	0.339	0.0335	0.0858	-0.0130
Cemented toilet	0.00	0.17	0.22	0.56	0.78	0.34	0.341	0.475	0.2625	0.3642	-0.1884
Soap	0.84	1.00	0.97	1.00	1.00	0.96	0.962	0.193	0.1105	0.0218	-0.5508
Insecticide spray	0.05	0.11	0.05	0.08	0.08	0.08	0.077	0.267	0.0653	0.2257	-0.0188
Mosquito net	0.03	0.03	0.11	0.14	0.44	0.15	0.148	0.356	0.2083	0.4985	-0.0866
Main mean of transportation											
Feet/walking	0.97	0.94	0.89	0.81	0.47	0.82	0.819	0.386	-0.2108	-0.0989	0.4468
Bicycle	0.03	0.06	0.08	0.11	0.33	0.12	0.121	0.327	0.1252	0.3367	-0.0463
Car/motorbike	0.00	0.00	0.03	0.08	0.19	0.06	0.060	0.239	0.1696	0.6670	-0.0426
											
Number of households	37	36	37	36	36	182					

^a ^Reports the average number of people per sleeping room in the respective wealth quintile

^b ^Computed score for one additional person per sleeping room: -0.0232

The first components of the eigenvectors explained 16.1% of the variability in asset possession in the first and 17.7% in the second survey. In both surveys, highest values were assigned to the possession of a video (standardized asset scores of 0.85 and 1.26, respectively). Lowest values were assigned to having only wooden walls in the first survey (-0.97) and to having no soap in the second survey (-0.55).

### Longitudinal analysis of relative SEP

**Figure 2:**
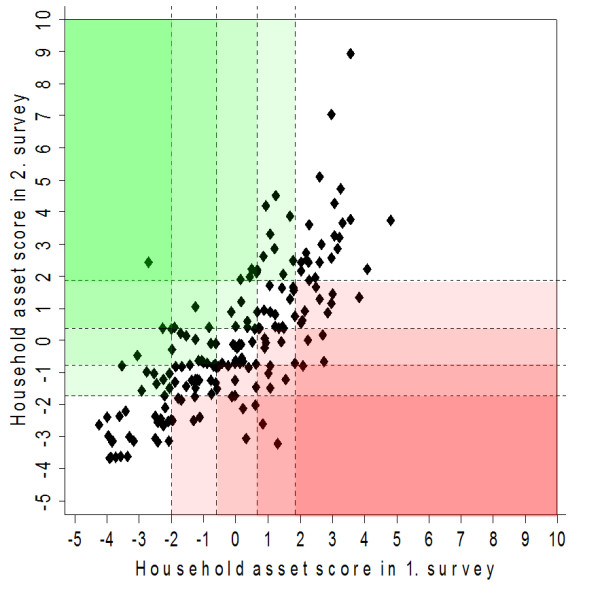
**Scatter plot showing the individual household asset scores of the first survey **(August **2002) against the scores of the second survey (late 2003/early 2004), with dotted lines vertical to the axis defining the respective wealth quintile cut-off points**.

Plotting the calculated individual household scores of the first survey against the scores of the second survey in a scatter plot reveals the dynamics in relative SEP of the studied households. In Figure 2, households with improved relative SEP over the length of study were plotted closer to the upper left corner (greenish) and households with worsened relative SEP ended up in the lower right corner (reddish). In contrast, households which experienced a relatively stable socioeconomic period, be it on a high level (upper right corner) or on a low level (lower left corner), are situated close to the diagonal. According to this reasoning, increasingly reddish or greenish areas in Figure 2 indicate higher household mobility with respect to their relative SEP. The dotted lines that are vertical to the axis and define the different rectangles represent the wealth quintile cut-off points in the respective surveys.

Table 4 shows that the relative SEP of 88 households (48.4%) changed between the first and second surveys. Overall, between the two time periods, 26 households (14.3%) rose or dropped by more than one quintile and twelve households (6.6%) could be classified as 'better-off' and 14 households (7.7%) as 'worse-off'.

**Table 4:** Cross tabulation of the number of households in the different wealth quintiles in the two surveys, August 2002 and late 2003/early 2004.

	Wealth quintile in the 1^st ^survey					
		
Wealth quintile in the 2^nd ^survey	Most poor	Very poor	Poor	Less poor	Least poor	Total
**Least poor**	1	0	5	8	22	36
**Less poor**	1	3	6	16	10	36
**Poor**	2	11	15	6	3	37
**Very poor**	8	16	7	4	1	36
**Most poor**	25	6	4	2	0	37
**Total**	37	36	37	36	36	182

### Characteristics of 'better-off', 'even' and 'worse-off' households

Tables 5 and 6 present an overview of the households' socioeconomic fates and other characteristics, which were reported in the two surveys. We found no statistically significant associations between the socioeconomic fates of households and reported characteristics of their respective heads of households, households' compositions and accessibility, migrational or natural population changes, severe cases of illness, and important economic activities in the respective village of residence. The only significant associations found were related to problems reported since the beginning of the armed conflict. 'Even' households mentioned more often health-related problems than 'worse-off' or 'better-off' households (p = 0.022). Additionally, 'better-off' households complained more often about the interruption of public services (p = 0.050) and the lack of food (p = 0.001) than their 'even' or 'worse-off' counterparts.

**Table 5:** Household characteristics at the onset of the armed conflict in Côte d'Ivoire (first survey, August 2002) and households' socioeconomic fate during the armed conflict.

Characteristics	Socioeconomic fate			
		
	'Worse-off'	'Even'	'Better-off'	p-value
Head of household (% of households with the same socioeconomic fate)				
Sex: male	92.9	89.7	91.7	
Sex: female	7.1	10.3	8.3	0.999*
Education: no	78.6	55.8	66.7	
Education: primary	21.4	32.1	25.0	
Education: higher	0.0	12.2	8.3	0.559*
Occupation: farmer	100.0	85.3	100.0	
Occupation: merchant	0.0	2.6	0.0	
Occupation: retiree	0.0	5.8	0.0	
Occupation: teacher	0.0	1.9	0.0	
Occupation: others	0.0	4.5	0.0	0.999*
Household composition (averages based on counts)				
Average no. of inhabitants	10.6	9.6	8.3	0.872**
Average no. of males	4.7	5.0	4.25	0.804**
Average no. children (< 16 years)	5.6	4.8	3.8	0.804**
Accessibility (% of households with the same socioeconomic fate)				
Gravel road	7.1	35.3	25.0	
Minor road	50.0	26.3	33.3	
Main road	42.9	38.5	41.7	0.162*
Located in village with the following economic activities (% of households with the same socioeconomic fate)				
Subsistence farming	100.0	100.0	100.0	1.000*
Agricultural production	100.0	100.0	100.0	1.000*
Trade	42.9	40.4	33.3	0.899*
Fishing	28.6	40.4	41.7	0.765*
Wood processing	28.6	14.7	16.7	0.765*
Artisanry	0.0	4.5	8.3	0.765*

* Based on Fisher's exact test

** Based on Kruskal-Wallis test

**Table 6:** Household characteristics after the armed conflict in Côte d'Ivoire (second survey, late 2003/early 2004) and households' socioeconomic fate during the armed conflict.

Characteristics	Socioeconomic fate			
		
	'Worse off'	'Even'	'Better off'	p-value
Head of household (% of households with the same socioeconomic fate)				
Sex: male	85.7	87.8	91.7	
Sex: female	14.3	12.2	8.3	0.887*
Education: no	71.4	60.3	50.0	
Education: primary	28.6	24.4	41.7	
Education: higher	0.0	15.4	8.3	0.390*
Occupation: farmer	100.0	96.2	91.7	
Occupation: merchant	0.0	3.2	8.3	
Occupation: retiree	0.0	0.0	0.0	
Occupation: teacher	0.0	0.0	0.0	
Occupation: others	0.0	0.6	0.0	0.391*
Household composition (averages based on counts)				
Average no. of inhabitants	8.6	9.0	8.9	0.995**
Average no. of males	4.9	4.6	4.0	0.695**
Average no. children (< 16 years)	5.5	4.5	6.6	0.502**
Accessibility (% of households with the same socioeconomic fate)				
Gravel road	7.1	35.3	25.0	
Minor road	50.0	26.3	33.3	
Main road	42.9	38.5	41.7	0.162*
Located in village with following economic activities (% of households with the same socioeconomic fate)				
Subsistence farming	100.0	100.0	100.0	1.000*
Agricultural production	100.0	100.0	100.0	1.000*
Trade	42.9	60.9	66.7	0.380*
Fishing	57.1	39.1	33.3	0.380*
Wood processing	0.0	0.0	0.0	1.000*
Artisanry	0.0	0.0	0.0	1.000*
Migrational change during the armed conflict (% of households with the same socioeconomic fate)				
< 0	21.4	24.4	50.0	
0	64.3	64.1	41.7	
> 0	14.3	11.5	8.3	0.383*
Natural population change during the armed conflict (% of households with the same socioeconomic fate)				
Births: 0	78.6	83.3	83.3	
Births: 1	21.4	13.5	16.7	
Births: 2	0.0	3.2	0.0	0.812*
Deaths: 0	64.3	62.8	75.0	
Deaths: 1	28.6	24.4	25.0	
Deaths: >1	7.1	12.8	0.0	0.835*
Heavy cases of illness during the armed conflict (% of households with the same socioeconomic fate)				
0	21.4	37.8	41.7	
1	28.6	33.3	16.7	
> 1	50.0	28.8	41.7	0.376*
Reported problems during the armed conflict (% of households with the same socioeconomic fate)				
Socioeconomic difficulty	50.0	72.4	83.3	0.134*
Health-related problems	21.4	52.6	25.0	0.022*^#^
IDPs/refugees	0.0	10.9	8.3	0.568*
Public services interrupted	0.0	12.8	33.3	0.050*^#^
Lack of food	14.3	26.9	75.0	0.001*^#^
Insecurity and threats	0.0	12.8	0.0	0.285*

* Based on Fisher's exact test

** Based on Kruskal-Wallis test

# Significant at 95% significance level

**Figure 3:**
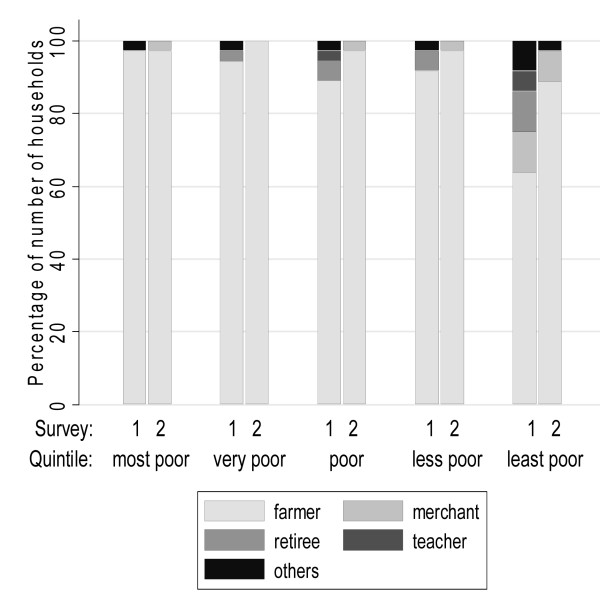
**Stack bar diagram of the main occupations of the respective heads of households, stratified by survey and wealth quintiles**.

Comparison of the households' characteristics between the first and the second survey, irrespective of the households' socioeconomic fates, indicated a change in livelihood strategies. Significantly more heads of household were mainly engaged in farming in late 2003/early 2004 than in 2002 (McNemar's χ² test: p < 0.001). At the same time, the number of merchants increased and the number of teachers decreased, but neither trend was statistically significant (McNemar's χ² test: p = 0.527 and p = 0.083, respectively). However, the number of retirees (McNemar's χ² test: p = 0.003) and all other occupations (McNemar's χ² test: p = 0.008) significantly decreased (see Tables 5 and 6 and Figure 3). These findings are supported by reports of the unchanged importance of the agricultural sector in the villages of residence and increased importance of trade (McNemar's χ² test: p < 0.001), whereas wood processing (McNemar's χ² test: p < 0.001) and artisanry (McNemar's χ² test: p = 0.005) completely disappeared and only fishing remained (McNemar's χ² test: p = 0.895).

### Longitudinal analysis of equity shifts

Table 7 summarizes the MP/LP ratios and the CIs. As indicated by MP/LP ratios >1, possession of land and house, higher numbers of people per sleeping room, low quality housing, cooking with wood, poor sanitation and walking as the main mean of transportation are 'bads' and are observed more often in the 'most poor' than in the 'least poor' households. Conversely, good quality housing, cooking with coal, possession of electronic devices, good sanitation and protective measures and mechanical means of transportation are 'goods' and are observed more often in the 'least poor' than in the 'most poor' households.

**Table 7:** Equity indicators of the studied households in the first (August 2002) and second (late 2003/early 2004) survey.

	MP/LP*		CI** [Conf. Int.***]			
		
Asset variable	August 2002	Late 2003/early 2004	August 2002		Late 2003/early 2004	
Possession of land	1.21	1.13	-0.03	(-0.06, -0.01)	-0.02	(-0.05, 0.00)
Possession of house	1.09	1.13	-0.01	(-0.03, 0.01)	-0.02	(-0.05, 0.00)
People per sleeping room	1.55	1.26	-0.12	(-0.18, -0.06)	-0.08	(-0.15, 0.00)
Type of wall						
Wood	n.s.^a^	n.s.^a^	-0.97	(-1^c^, 0.32)	-0.72	(-1^c^, 0.08)
Mud/clay	n.s.^a^	n.s.^a^	-0.44	(-0.52, -0.35)	-0.64	(-0.77, -0.52)
Cement	0.08	0.16	0.25	(0.21, 0.30)	0.23	(0.19, 0.27)
Type of roof						
Thatch	n.s.^a^	n.s.^a^	-0.75	(-1^c^, -0.49)	-0.74	(-1^c^, -0.48)
Tiles	0.96	0.96	0.26	(-0.12, 0.64)	-0.06	(-0.37, 0.26)
Corrugated sheets	0.39	0.47	0.12	(0.07, 0.17)	0.13	(0.08, 0.17)
Energy source for cooking						
Wood	1.06	1.09	-0.01	(-0.03, 0.00)	-0.01	(-0.04, 0.01)
Coal	0^b^	0^b^	0.57	(-0.09, 1^c^)	0.40	(-0.27, 1^c^)
Electronic devices						
Electricity in house	0.05	0.18	0.41	(0.36, 0.45)	0.30	(0.24, 0.36)
Refrigerator	0^b^	0^b^	0.72	(0.25, 1^c^)	0.87	(0.40, 1^c^)
Radio	0.38	0.22	0.19	(0.14, 0.25)	0.29	(0.24, 0.35)
Television	0^b^	0^b^	0.66	(0.55, 0.77)	0.65	(0.54, 0.77)
Ventilator	0^b^	0^b^	0.70	(0.42, 0.99)	0.77	(0.51, 1^c^)
Video	0^b^	0^b^	0.96	(-0.88, 1^c^)	0.96	(-0.02, 1^c^)
Sanitation infrastructure and personal protection						
No toilet	6.58	11.40	-0.41	(-0.49, -0.33)	-0.34	(-0.39, -0.29)
Uncemented toilet	2.89	0.39	-0.25	(-0.52, 0.02)	0.17	(-0.02, 0.35)
Cemented toilet	0.13	0^b^	0.37	(0.31, 0.42)	0.46	(0.37, 0.55)
Soap	1.03	0.84	0.00	(-0.01, 0.01)	0.03	(0.01, 0.05)
Insecticide spray	0.28	0.65	0.38	(0.19, 0.57)	0.11	(-0.22, 0.45)
Mosquito net	0.42	0.06	0.15	(-0.01, 0.31)	0.55	(0.33, 0.76)^#^
Main mean of transportation						
Feet/walking	2.00	2.06	-0.14	(-0.18, -0.10)	-0.12	(-0.15, -0.08)
Bicycle	0^b^	0.08	0.62	(0.36, 0.88)	0.45	(0.23, 0.67)
Car/motorbike	0^b^	0^b^	0.43	(0.16, 0.70)	0.68	(0.33, 1^c^)

* Most poor-least poor ratio (adjusted for the difference in total number of households in the two quintiles)

** Concentration index

*** 95% confidence interval of CI

# Statistically significant equity shifts

a Not specified as division by zero is mathematically not defined

b Dividend is 0, indicating that no household of the most poor quintile owned the respective asset

c The calculated confidence interval by using the "convenient regression method" includes values larger than 1 or smaller than -1. As CI is only defined in the interval (-1,1), the values in the table are adjusted accordingly.

The CIs largely confirmed the findings from MP/LP ratios by assigning negative values to 'bads' and positive values to 'goods'. Neither indicator demonstrates exceptional equity shifts between the two surveys. The only statistically significant shift was an increased concentration of mosquito nets in wealthier households, as shown by the 95% confidence intervals of the CIs for 2002 and 2004 (see endnote 4).

## Discussion

Micro-level data on relative SEP of households and its dynamics in the context of an armed conflict have been presented. Our data stem from two cross-sectional household surveys, the first carried out just before, and the second one and a half years after the outbreak of an armed conflict in Côte d'Ivoire.

Our secondary data analysis revealed consistency of the underlying data, illustrated by the high recapture rate of households (89.7%), and the accuracy of population data (potential misreporting of only 0.5% of the original population in the second survey). The reliability of the data, methods, and the constructed wealth index was confirmed by the algebraic sign assigned to each asset. The only exceptions were the negative values assigned to the variables for house possession and land tenure. However, the absences of house and land ownership could be interpreted as proxies for employed work, which plausibly could be associated with higher wealth in the rural setting under investigation. In fact, households which did not possess land or housing were significantly more often engaged in non-agricultural work than other households (p < 0.001 for land and housing in both surveys). This exception has also been identified, and has been explained accordingly, in other rural settings when utilizing the same asset-based approach for wealth assessment (see for example reference [55]).

Despite the exceptional circumstances and the fact that 130 households (71.4%) mentioned socioeconomic difficulties, our analyses revealed weak socioeconomic dynamics with only every seventh household (14.3%) being labeled 'worse-off' or 'better-off' between the two surveys. Furthermore, no dramatic equity shifts were found. Total asset possession of all households varied little over the length of the study, with extremes of -15% for disposing of working cemented toilets and +13% for not having a toilet at all (see Tables 2 and 3 and endnote 4).

An attrition analysis of the 21 households which were interviewed in the first survey but could not be revisited in the second survey (see Figure 1) gave no indication of a selection bias, as the excluded households were symmetrically distributed over the wealthier and poorer wealth quintiles. Furthermore, there was no significant difference in the reported characteristics between those households included and those lost to follow-up. While we cannot exclude the possibility that households lost to follow-up lost all their assets or were banished as a consequence of the armed conflict, by the same token it is possible that they became wealthier or could improve their social status and decided to migrate for these reasons. Reports from those households that remained in the area and participated in the follow-up survey revealed that among households lost to follow-up, 5 out of 21 (23.8%) migrated to rebel-controlled areas and another 5 (23.8%) moved to government-controlled territories. This anecdotal evidence suggests that motivations and causes to migrate were diverse. A possible bias due to the operation of humanitarian aid projects in the area is also considered unlikely as the second round of household interviews took place before these humanitarian activities were fully implemented. Furthermore, the humanitarian aid projects were targeted on community rather than household or individual level. Hence, it is plausible that the observed dynamics are neither a matter of systematic asset in- or outflow nor a simple methodological artifact. Rather, the fluctuations in relative SEP seem to be the result of a limited reallocation of the always present assets among the studied households, without remarkable equity shifts.

The weak socioeconomic dynamics may at least partly explain why, except for the self-reported problems encountered since the beginning of the armed conflict, no significant associations were identified between the households' socioeconomic fate and other characteristics. As shown in Table 6, 'even' households mentioned significantly more often health-related problems than 'worse-off' or 'better-off' households. This finding is difficult to interpret, but the fact that 'better-off' households complained more often about the interruption of public services and the lack of food than their 'worse-off' or 'even' counterparts may reflect that they are not used to have any difficulties in these domains. Previous research in the same study area also observed that schoolchildren from wealthier households complained more often about suffering from disease symptoms than their less wealthy but equally healthy peers [46]. This pattern was identified in other epidemiological settings as well [56,57] and explained with higher expectations of the 'better-off', which makes them more sensitive to distress and consequently also more likely to complain.

Our findings of changed livelihood strategies are consistent with results from other reports [37,44]. One study carried out in central, north and west Côte d'Ivoire investigated the effect of the same armed conflict on human resources and the functioning of the health system. Significant reductions of well-trained staff in both the public and the private sector were observed, along with a collapse of the health system and other components of public infrastructure [37]. In the current investigation, we recognized a significant return to primary production of the interviewed heads of households. Nevertheless, we found no statistically significant associations between a household's socioeconomic fate and its main occupation or other important economic activities in the village of residence.

However, several particularities of the study design and the methodological approach applied may have influenced the outcome of the analyses. First, our sample size is small and therefore confidence intervals are large. This fact may partially explain why most changes and associations were insignificant. Second, due to the potentially low level of wealth before the outbreak of the armed conflict, even a small change in asset possession may have been sufficient for a change in the wealth quintile classification. Furthermore, a certain degree of elusiveness was inevitable as we had to rely on a secondary analysis of self-reported information and could not investigate all relevant indicators. For example, the insignificant associations between the households' socioeconomic fates and certain household characteristics may simply highlight that the usual characterization of households (e.g. age, sex, education, occupation, changes in household composition, accessibility or illness) does not capture the most important factors of what made households 'better-off' and 'worse-off' after the armed conflict. Local power structure, ethnic group, family feuds, and rivalry may be even more important than usual during an armed conflict, as implied by informal comments of the study participants regarding trafficking of drugs and other illegal and stolen goods (see endnote 6).

Certain weaknesses in the study design are partly owed to the very nature of the study, since working in conflict areas is a challenging task with many unforeseeable events. Access to, and movement of, populations may lead to a selection bias, as discussed above. Furthermore, war-related research cannot rely on any experimental design and instead must depend upon observational studies, which are limited in their ability to address causality. The timing of any war-related research is particularly difficult. Social tensions and sporadic physical violence may precede and only slowly wane after a fully fledged armed conflict. Hence, it is difficult to obtain reliable 'pre-conflict' baseline and 'post-conflict' follow-up data. Often, it is uncertain whether the observed dynamics were just the near completion of a process that already began years ago (i.e. before baseline survey) or whether many serious consequences might still occur in the future (i.e. after follow-up survey) (see endnote 1).

Methodologically, household asset-based approaches for estimating relative SEP have proven to be valid in rural Côte d'Ivoire, as well as in other African settings [58]. This is especially true for the method applied here, which has been adapted and widely used for health surveys in various locations including the present study area [41,42,46-49]. Hence, the methodological approach seems to be adequate for analyzing the effects of an armed conflict on relative SEP of households. However, a study from southeast Nigeria found some indication that the reliability of asset-based wealth indices is only moderate [59], so we cannot exclude the possibility that a few of the identified changes in relative SEP were simply due to measurement errors.

Asset-based socioeconomic indices were calculated to estimate the households' relative SEPs in "a pragmatic response to data constraints" [50]. Asset possession was measured only as a binary socioeconomic indicator, irrespective of quantity or quality. Assets were not valued at current monetary market prices, but weighted with 'raw' asset factors originating from PCA. These 'raw' asset factors were re-calculated for both surveys, which may seem quite arbitrary, even though they changed only slightly in our study. However, at times of an armed conflict, relative importance of assets may change rapidly. Therefore, we adopted an approach with flexible weights (see endnote 7). Nevertheless, computed household scores represented ordinal values, which allowed for ranking the involved households, as opposed to cardinal values (e.g. monetary values), which would also provide information about changes in absolute wealth.

The idea that "(asset) weights should be allowed to vary over time" [60] is in line with a statement by Sahn and Stifel (2000). However, in their aggregate analysis of poverty over time and across African countries, they used fixed weights based on the results of pooled wealth indicators. They evaluated socioeconomic dynamics by setting fixed poverty lines at 25% and 40% as anchor points, and subsequently counted households to see whether more or less fall below these anchor points at certain places or points in time [60]. Hence, their approach is more rigid, but allows for direct temporal and regional comparison.

The focus on relative wealth dynamics seems appropriate as people tend to evaluate their living conditions by comparing their current with their previous circumstances or with the circumstances of people in their surroundings. This fact may also explain the findings of Goodhand (2003) that "absolute measures of poverty may be less significant (...) as triggers to violence" and that "transient poverty is likely to have a more significant influence on the dynamics of war and peace than chronic poverty" [1].

However, given the analytical approach, which is self-contained and not open as opposed to income or consumption studies, households have limited options for mobility. Only households in intermediate quintiles can truly rise and fall in the frame set by asset scores and wealth quintiles. In general, it is possible that asset-based wealth indices are more stable than other SEP indicators (e.g. absolute poverty in monetary terms, income or consumption). In times of economic hardship, households may draw upon other resources before selling their assets. Likewise, in prosperous periods, it may take some time until economic success is reflected in asset possession and an associated wealth index.

As substantial negative consequences of the armed conflict in Côte d'Ivoire are demonstrated in other micro- and macro-level reports, and even by some of our own indicators, it is likely that our PCA-based analysis failed to detect at least some of the socioeconomic dynamics and associated predictors. Hence, it could be worthwhile to use a more comprehensive livelihood framework to assess the impact of armed conflicts in future investigations (see endnote 8). Furthermore, it would be useful to compare our results with other war-torn as well as peaceful settings in order to get a better idea about the threats and opportunities imposed on households by armed conflict as well as to further verify causality. In fact, high-quality data for comparison might be readily available from a growing number of demographic surveillance systems (DSS; see http://www.indepth-network.org; accessed 23 August 2010) or the comparatively new Household in Conflict Network (see http://www.hicn.org; accessed 23 August 2010).

In conclusion, we emphasize that more micro-level research on the measurement of SEP in low-income countries as well as on the impact of armed conflict and war is warranted. In general, methods to assess and meaningfully interpret longitudinal micro-level wealth data should be further developed. Recent methodological reviews demonstrated that all actual approaches for measuring SEP in low-income countries have their drawbacks. Furthermore, the results of the different methods showed only limited agreement and have restrictions for further processing [59,61-64]. Along with previous research undertaken in conflict zones elsewhere in Africa [37,65-76], the present study can be considered as additional evidence of the feasibility of research even in troubled times and zones. Knowledge about the profiles of households that are more resilient to armed conflict could help to better prevent and/or alleviate adverse conflict-related and increasingly civilian-borne socioeconomic effects. Consequently, we would like to reinforce Tam and colleagues' "call to arms" [77] in order to boost research related to armed conflict and war.

## Competing interests

The authors declare that they have no competing interests.

## Endnotes

1. For the purpose of this paper, the term "armed conflict" refers to the warlike events in Côte d'Ivoire between August 2002 and early 2004, and can be defined as a "contested incompatibility, which concerns government and/or territory where the use of armed force between two parties, of which at least one is the government of a state, results in at least 25 battle-related deaths" [78]. However, this definition is not comprehensive as it does not include, for example, conflict between non-state actors or social violence. Furthermore, one should be aware of the fact that "the distinction between war, predatory violence, and crime are becoming increasingly blurred" [1]. In our own analyses, we looked at the armed conflict as defined above, and considered the social tensions and sporadic physical violence, which preceded and followed the fully-fledged armed conflict, only in the interpretation of our findings in the discussion.

2. For further reading see references [37,40-43,45-49,79-90].

3. The project "Programme d' appui de la DDC à la réconciliation en Côte d'Ivoire" was supported by the Swiss Agency for Development and Cooperation (SDC) from 2003-2006. Using the concept of valorizing both research results and social trust earned in longstanding scientific partnerships, it comprised activities in three socio-cultural zones of Côte d'Ivoire to foster reconciliation and community cohesion in the period after the armed conflict. For this sociopolitical motive, the project also drew on the conservation of the Taï National Park and food security based on production, processing and post-harvest storage of yam and cassava (for further reading see references [45,91]).

4. For more details on operational results, households' demographic profiles and a comprehensive secondary analysis of the impact of the armed conflict on risk factors for malaria and neglected tropical diseases (NTDs), the reader is referred to reference [28].

5. The book by O'Donnell and colleagues (2008) can be downloaded at http://go.worldbank.org/LVSSZJX9O0 (accessed 23 August 2010). It is an aggregation and revision of the technical notes, originally put forth by the World Bank's Poverty Reduction and Economic Management (PREM) web site (http://www1.worldbank.org/prem/poverty/health/wbact; accessed 26 September 2007; however, no longer accessible).

6. We have anecdotal evidence of "mercenaries carrying away truckloads of corrugated sheets and electronic goods". Consequently, some households seemed to hesitate to invest in new assets because of the fear of looting and because increased asset possession could pose an additional health threat to household members due to violence. While we cannot prove looting in the surveyed area, we assume that its frequent occurrence elsewhere also had a backlash on our findings.

7. In fact, the household rankings barely changed, whether we used 'raw' asset factors based only on first survey data for weighting both surveys (Spearman's rank correlation coefficient for first survey indices: 1.00, p < 0.001; and 0.99, p < 0.001 for second survey indices), or 'raw' asset factors based only on second survey data for weighting both surveys (0.99, p < 0.001; and 1.00, p < 0.001), or 'raw' asset factors based on pooled data (0.98, p < 0.001; and 0.99, p < 0.001). These findings are also in line with a paper by Sahn and Stifel (2000) (see their Note 17 in reference [60]).

8. See, for example, the comprehensive livelihood framework elaborated by the United Kingdom's Department for International Development (DFID), which includes the relevant context of vulnerability, the different livelihood assets and strategies, and the transforming structures and processes [92].

## Authors' contributions

TF analyzed and interpreted the data and drafted the manuscript. ABT conceptualized and designed the study and was also involved in data analysis, data interpretation and article drafting. GR, CAA, OG and EKN contributed to the study conceptualization and design as well as data acquisition. DdS assisted in data analysis and interpretation. JU supervised TF and was involved in conceptualization and design of the study, data analysis and data interpretation. All authors were involved in critical review and revision of the manuscript, and read and approved its final version for publication.
